# A population description of CULAs using combined documentation of compound phenotypes according to the Oberg-Manske-Tonkin classification

**DOI:** 10.1016/j.jpra.2025.03.023

**Published:** 2025-04-05

**Authors:** Feikje Julia ten Cate, Margriet Harmke Maria van Doesburg

**Affiliations:** Amsterdam University Medical Center (AUMC), Department of Plastic, Reconstructive and Hand surgery, Meibergdreef 9, 1105 AZ, Amsterdam, The Netherlands

**Keywords:** Classification strategy, CULA, OMT classification, Oberg-Manske-Tonkin

## Abstract

**Background/Purpose:**

Congenital upper limb anomalies (CULAs) exhibit a wide range of manifestations. For this reason, the Oberg-Manske-Tonkin (OMT) classification was introduced to achieve etiologically correct and universal classification. Combined use of codes has been advocated to prevent the loss of important phenotypic information in compound phenotypes. Therefore, the aim of our study was to present a description of our population over the last 10 years, using the most recent (2020) version of the OMT and combined documentation when necessary.

**Methods:**

All patients who visited our tertiary referral hospital between 2010 and 2020 were analyzed retrospectively and classified using the OMT. Combined registration was allowed in compound phenotypes and combinations were analyzed.

**Results:**

Overall, 797 patients were included in our registry. Most anomalies were classified in the malformations group and 9.5% required combined documentation; 13.5% of the possible combinations were observed in opposite extremities. Syndactyly, polydactyly (radial and ulnar), and camptodactyly were the most common diagnoses. The most frequently observed recurring combinations were brachydactyly and clinodactyly, ulnar longitudinal deficiency and simple syndactyly, and simple syndactyly and clinodactyly.

**Conclusions:**

Approximately one-tenth of our population required combined documentation. When using compound documentation, we recommend providing information regarding the presence or absence of a combination in the same extremity. Failing to do so could lead to loss of equally important phenotypic information as disregarding combinations would. Epidemiologic registries such as ours, allow outcome comparison and provide information for research on underlying etiologies. Additionally, our research focused on the analysis of combinations of codes.

Level of evidence: 3

## Introduction

Congenital upper limb anomalies (CULAs) are relatively uncommon, with an incidence of approximately 27 per 10,000 births.^1^ Given the wide spectrum of phenotypes displayed by these children, a uniform and comprehensive classification system is essential. The Oberg-Manske-Tonkin classification (OMT) is embraced by the International Federation of Societies for Surgery of the Hand (IFSSH) for the classification of CULAs because it allows for a more appropriate categorization of these anomalies, according to most recent scientific advances,^2^ than the previously used Swanson classification.^3^

Several epidemiologic studies have been conducted since its introduction. However, in 2018 Baas et al.^4^ noticed inconsistencies in combined documentation as most studies only registered the main anomaly per arm. Their study on the necessity of combined documentation found that a fifth of their patients required a combination of codes. Disregarding these compound phenotypes could lead to the loss of important information and hinder epidemiologic research and fair outcome comparison. Additionally, when compound phenotypes are well documented, subgroups within a population can be identified and could indicate an underlying etiology.

The purpose of our study was to provide a description of the CULA population at our tertiary referral hospital using the most recently modified (2020) version of the OMT classification,^5^ with combined documentation if required. Our second aim was to describe and analyze the code combinations registered in compound phenotypes.

## Materials and Methods

This study adhered to the strengthening the reporting of observational studies in epidemiology (STROBE) guidelines for observational research

### Sample

An observational cohort study was conducted. Data from all patients who visited our tertiary hospital for congenital hand or upper limb anomalies between April 2010 and April 2020 were reviewed retrospectively. Patients suitable for inclusion were all patients who visited the outpatient clinic for a first consultation or for follow-up. Patients with anomalies due to trauma, acquired anomalies, anomalies of the feet, and trigger fingers were excluded. Patient characteristics, medical history, location of the affected limb(s), and genetic test results were obtained. Radiographs and clinical photographs were acquired if they were available.

### Classification strategy

All patients were reviewed by hand and classified by the first author using the 2020 update of the OMT classification.^5^ In the event of an ambiguity, a pediatric hand surgeon was consulted. Syndrome diagnosis was acquired from medical records, including genetic test results if available.

Multiple codes were registered in case of compound phenotypes. Bilaterally identical OMT diagnoses were counted and analyzed individually.

### Combination analysis

The number of times a combination of two or more different codes was necessary was registered. In case of more than two different codes in one patient, all possible combinations were analyzed and counted. Additionally, information regarding the presence of a combination in the same extremity was documented.

## Results

### Registry characteristics

Between April 2010 and April 2020, a total of 797 patients were eligible for inclusion. Because patients could get more than one OMT classification code, a total of 874 OMT diagnoses were recorded. Overall, 428 OMT diagnoses were registered bilaterally and 446 unilaterally (left/right, 275/171; Table 1). Table 1 shows the number of times a certain code was seen unilaterally or bilaterally in a unique patient. Most anomalies were categorized in the malformations group. The first and second most commonly registered anomalies were ulnar and radial polydactyly, followed by cutaneous syndactyly and camptodactyly. In 84 patients (10.5% of the population), a syndrome was registered, with VACTERL association, Poland syndrome, and Greig Cephalopolysyndactyly being the most frequently observed (Table 2).

**Table 1:** Distribution of CULAs according to the OMT classification, anomalies seen ≥25 times in bold font. Underlined numbers represent the total amount per main OMT group

OMT Diagnosis	Left	Right	Bilateral	Total
**I. Malformations**	238	156	319	713
IA1i (Brachymelia)	0	1	0	1
IA1iia (Symbrachydactyly spectrum-Poland syndrome)	3	1	0	4
IA1iib (Symbrachydactyly spectrum-whole limb excluding Poland syndrome)	1	1	0	2
IA1iiib (Transverse deficiency-segmental)	9	5	2	16
IA2i (Radial longitudinal deficiency)	6	5	6	17
IA2ii (Ulnar longitudinal deficiency)	5	4	2	11
IA2iv (Radiohumeral synostosis)	1	0	0	1
IA2v (Radioulnar synostosis)	8	6	5	19
IA2vi (Congenital dislocation of radial head)	5	1	3	9
IA2vii (Forearm hemiphyseal dysplasia, radial-Madelung deformity, or ulnar)	5	6	33	**44**
IA4ia (Sprengel deformity)	3	2	0	5
IB1i (Brachydactyly)	7	3	17	**27**
IB1ii (Symbrachydactyly)	24	13	4	**41**
IB1iii (Transverse deficiency)	8	7	1	16
IB1iv (Cleft hand-split hand foot malformation)	4	2	4	10
IB2i (Radial longitudinal deficiency, hypoplastic thumb)	9	9	19	**37**
IB2ii (Ulnar longitudinal deficiency, hypoplastic ulnar ray)	4	1	4	9
IB2iii (Radial polydactyly)	33	37	12	**82**
IB2iv (Triphalangeal thumb)	3	5	4	12
IB2iva (Triphalangeal thumb-five finger hand)	3	1	4	8
IB2v (Ulnar dimelia-mirror hand)	0	2	0	2
IB2vi (Ulnar polydactyly)	52	17	123	**192**
IB4ia (Cutaneous -simple-syndactyly)	27	9	34	**70**
IB4iia (Osseous-complex-syndactyly)	5	5	5	15
IB4iib (Clinodactyly)	10	4	28	**42**
IB4iid (Synostosis/symphalangism)	0	1	3	4
IB4iiia (Syndromic syndactyly; e.g., Apert hand)	0	0	3	3
IB4iiib (Synpolydactyly)	3	5	1	9
IB4iiic (Unspecified axis-complex-not otherwise specified)	0	3	2	5
**II. Deformations**	9	4	9	22
IIA (Constriction ring sequence)	9	4	9	22
**III. Dysplasias**	28	11	100	**139**
IIIA2i (Macrodactyly)	4	3	4	11
IIIA2ii (Aberrant flexor/extensor/intrinsic muscle)	1	0	0	1
IIIB1i (Hemangioma)	1	0	0	1
IIIB1ii (Vascular malformation)	1	0	0	1
IIIB4ii (Enchondromatosis)	2	0	0	2
IIIB4iii (Fibrous dysplasia)	0	0	1	1
IIIB4vi (Other skeletal tumorous conditions)	0	0	1	1
IIICia (Amyoplasia)	1	0	25	**26**
IIICib (Distal arthrogryposis)	1	1	23	**25**
IIICic (Arthrogryposis multiplex congenita-other)	0	0	8	8
IIICiia (Camptodactyly)	15	7	27	**49**
IIICiib (Thumb in palm deformity)	0	0	9	9
IIICiic (Isolated congenital contracture-other)	2	0	2	4
Total:	275	171	428	**874**

**Table 2:** Distribution of syndromic patients, represented in bold, are the top 3 most frequently documented syndromes

Syndrome	Frequency
A5. Bardet-Biedl	5
A6. Beals	1
A9. Catel-Manzke	1
A10. Cornelia de Lange	1
A12. Down	5
A14. Fancnoni pancytopenia	1
A15. Freeman Sheldon	1
A19. Greig cephalopolysyndactyly	**7**
A2. Apert	3
A21. Hemifacial microsomia (Goldenhar syndrome)	1
A22. Holt-Oram	2
A26. Leri-Weill dyschondrosteosis	6
A28. Moebius sequence	1
A31. Noonan	1
A32. Oculodentodigital dysplasia	5
A35. Pallister-Hall	1
A37. Pierre Robin	1
A38. Poland	**10**
A45. Split hand-foot malformation	2
A46. Thrombocytopenia absent radius	3
A47. Townes–Brock	1
A48. Trichorhinophalangeal	1
A49. Ulnar-mammary	2
A50. VACTERL association	**12**
B. Others: 16p11 deletion syndrome	1
B. Others: 22q11 deletion syndrome	1
B. Others: 4q-deletion syndrome	1
B. Others: Aarskog syndrome	1
B. Others: Branchio-oculo-facial syndrome	1
B. Others: Chitayat syndrome	1
B. Others: Ellis-van Creveld syndrome	1
B. Others: Frontomethafysair dysplasia	1
B. Others: Karsch–Neugebauer syndrome	1
B. Others: Vanishing white matter syndrome	1
Total	84

### Combinations

Among the 797 patients documented, 76 required more than one type of OMT code. Among those compound phenotypes, 65 required 2 different codes, 10 needed 3, and in 1 patient four different codes were registered (Table 3). In 17% (n = 13) of the patients with compound phenotypes, a syndrome was registered.

**Table 3:** Number of patients with different OMT codes

Total number of patients	797
1 OMT type	721
2 OMT types	65
3 OMT types	10
4 OMT types	1

From these 76 patients who required multiple codes, 61 possible combinations of two different codes could be deducted. Thirty-four (38.2%) of those were extracted from phenotypes with threeor four different OMT codes, the rest were observed in patients with only two different codes. The original combinations observed in patients with three or four codes are shown separately in appendix A. Among the 61 possible different combinations, 16 were documented repeatedly and 45 only once.

In total, a combination of two different codes was counted 89 times. Twelve (13.5%) of these combinations of 2 OMT codes, were in opposite extremities. For example: Ulnar longitudinal deficiency (IA2ii) on the right hand and simple cutaneous syndactyly (IB4ia) on the left hand.

Sixteen recurring combinations accounted for 49.4% of all combined OMT diagnoses (n = 44).

To preserve comprehensibility, only the most frequently registered recurring combinations are shown in Table 4. The most commonly registered combinations were brachydactyly combined with clinodactyly, ulnar longitudinal deficiency of the upper limb combined with simple syndactyly, and simple syndactyly combined with clinodactyly. For the complete table of combinations see appendix A.

**Table 4:** Most frequently observed recurring combinations

Combination of OMT Diagnoses	Frequency (total)	Unilaterally (i.e., same extremity)	As part of larger combination
IB1i (Brachydactyly) and IB4iib (Clinodactyly)	5	5	0
IA2ii (Ulnar longitudinal deficiency entire upper limb) and IB4ia (Simple-cutaneous syndactyly)	4	3	0
IB4ia (Simple-cutaneous-syndactyly) and IB4iib (Clinodactyly)	4	3	1
IB4ia (Simple-cutaneous-syndactyly) and IB1i (Brachydactyly)	3	3	1
IB4iia (Osseous-complex-syndactyly) and IB4ia (Simple-cutaneous-syndactyly)	3	2	0
IB4ia (Simple-cutaneous-syndactyly) and IB2vi (Ulnar polydactyly)	3	2	1
IA2i (Radial longitudinal deficiency) and IA2vi (Congenital dislocation of radial head)	3	2	0
IB2i (Radial longitudinal deficiency, hypoplastic thumb) and IB4iia (Osseous-complex-syndactyly)	3	3	0

## Discussion

Similar to previous epidemiologic studies,^4^^,^^6^, ^7^, ^8^ syndactyly, polydactyly (radial and ulnar), and camptodactyly were most frequently observed. Unlike other registries, ours contained a relatively high count of Madelung deformities. This is probably the result of special expertise regarding that anomaly in our tertiary referral center.

Only 9.5% of our population required multiple OMT codes as opposed to more than 20.5% in the study by Baas et al.^4^. Their study also found a higher prevalence of triphalangeal thumbs combined with radial polydactyly. The latter could be the result of the high prevalence of a genetic mutation causing this anomaly in the southern Netherlands,^9^ near their hospital.

Regarding recurring combinations, brachydactyly and clinodactyly, ulnar longitudinal deficiency and simple syndactyly, and simple syndactyly and clinodactyly were the most frequently observed in our center. The high incidence of the last combination is likely to have a causal relationship. Untreated syndactyly could hinder the proper growth of one of the digits in a child and subsequently resulting in clinodactyly. All patients with this code combination, had it in the same digits that were involved in the syndactyly.

### Strengths and limitations

The strength of our study lies in the use of the most recent version of the OMT and focus on combined documentation when necessary. Another strength of our study is that we provided a detailed description of the combinations observed.

Unlike previous studies, combinations of bilaterally identical OMT codes were analyzed individually to acquire a more accurate description of our population.

**Figure 1:**
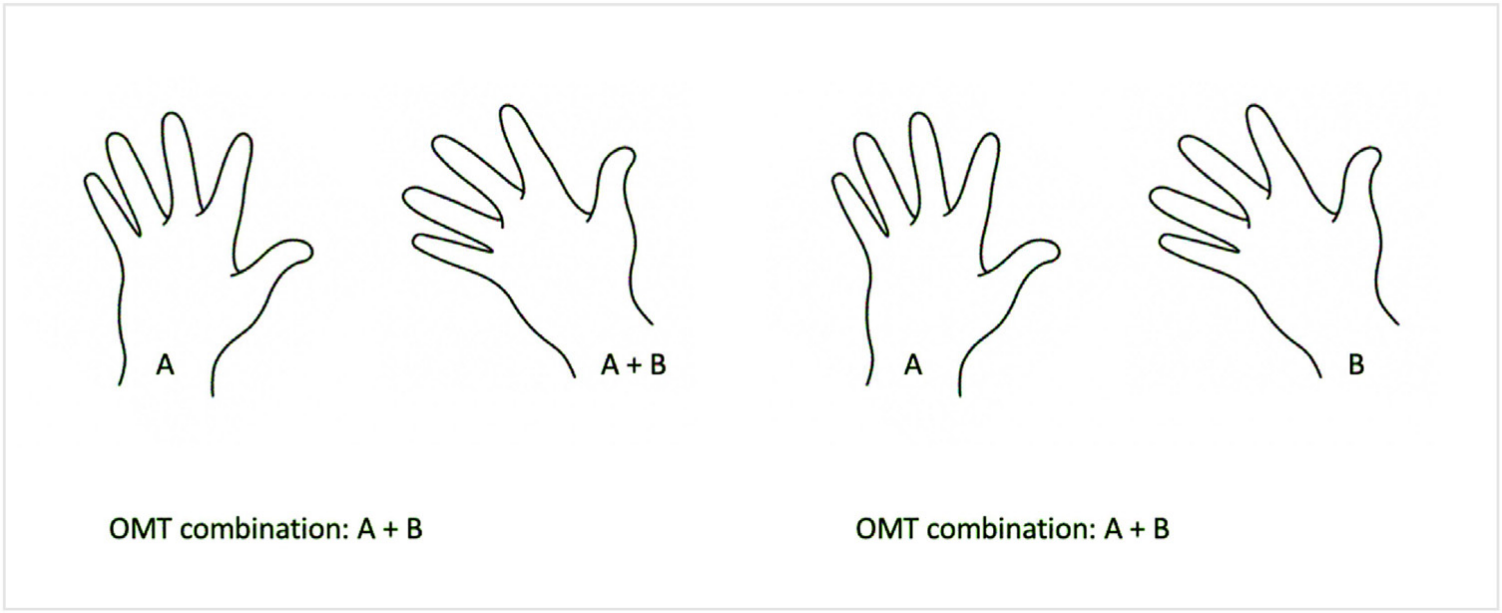
Ambiguous registration of OMT combination

Baas et al.^4^ demonstrated the necessity of combined documentation, but did not specify whether the combination was observed in the same extremity or solely in the same patient. With this approach, valuable information on the type of combination and its subsequent phenotype may be lost. For example: patient 1 has a combination of anomaly A on the left hand and anomaly A and B on the right hand. In this case, the combination of A and B is documented (Figure 1). This results in a correct description of the patient. If patient 2 has a combination of anomaly A on the left and B on the right hand, this combination is counted similarly. However, this patient has a very different phenotype and therefore different prospects regarding for example surgical outcome. In 13.5% of the times that a combination was counted, it was observed in the opposite extremity. Our study demonstrates that disregarding the information on laterality when documenting combined diagnoses, could lead to loss of potentially important information. Therefore, we recommend specifying if a combination occurred in the same limb or digit when registering using the OMT.

An important limitation of this study is that this study is a population description rather than a true epidemiologic analysis. Patients were seen in a tertiary referral center, therefore, no information on the prevalence or incidence can be provided. To provide these numbers, population data are required.

Another limitation is the retrospective design. Consequently, some classifications are based solely on the descriptions of physical examination by physicians, and several codes had no indication for an X-ray or medical photograph. Excluding these patients would have led to selection bias and an unreliable representation of our study population. Therefore, we decided to include patients regardless of the availability of these photographs.

In conclusion, we provided a detailed description of our population and thereby allow for comparison between facilities and provide information for research on underlying etiologies.

We agree with the proposition by Baas et al.^4^ to use combined documentation in the case of compound phenotypes and we additionally suggest including information on the specific location of the observed combination.

## Conflict of interest

The authors declare no potential conflicts of interest with respect to the research, authorship, and/or publication of this article.

## Appendix Supplementary materials

Image, application 1
