# Supplementary material for: A population description of CULAs using combined documentation of compound phenotypes according to the Oberg-Manske-Tonkin classification
Source: JPRAS Open. 2025 Apr 5;44:479–86. doi: 10.1016/j.jpra.2025.03.023 (PMC12099754; doi:10.1016/j.jpra.2025.03.023)
Supplement: Supplementary file 1 [file mmc1.docx]

Appendix A

| Combination of OMT Diagnoses | Frequency (total) | Unilateral (i.e. same extremity) | as part of larger combination | Type larger combination | Times part of syndrome | Type of syndrome |
| --- | --- | --- | --- | --- | --- | --- |
| IB1i (Brachydactyly) & IB4iib (Clinodactyly) | 5 | 5 | 0 |  | 1 | A48. Trichorhinophalangeal |
| IA2ii (ulnar longitudinal deficiency entire upper limb) & IB4ia (simple -cutaneous syndactyly) | 4 | 3 | 0 |  |  |  |
| IB4ia (simple -cutaneous- syndactyly) & IB4iib (Clinodactyly) | 4 | 3 | 1 | IB4ia & IB4iib & *IB1iv (Cleft hand)* |  |  |
| IB4ia (simple -cutaneous- syndactyly) & IB1i (Brachydactyly) | 3 | 3 | 1 | IB4ia & IB1i & *IB2i (Radial longitudinal deficiency, hypoplastic thumb)* |  |  |
| IB4iia (Osseous -complex- syndactyly) & IB4ia (Simple -cutanous- syndactyly) | 3 | 2 | 0 |  |  |  |
| IB4ia (simple -cutaneous- syndactyly) & IB2vi (Ulnar polydactyly) | 3 | 2 | 1 | IB4ia & IB2vi & IB2iii (Radial polydactyly) |  |  |
| IA2i (Radial longitudinal deficiency) & IA2vi (Congenital dislocation of radial head) | 3 | 2 | 0 |  | 1 | A10. Cornelia de Lange |
| IB2i (Radial longitudinal deficiency, hypoplastic thumb) & IB4iia (Osseous -complex- syndactyly) | 3 | 3 | 0 |  | 1 | A45. Split hand-foot malformation |
| IB2iii (radial polydactyly) & IB2vi (ulnar polydactyly) | 2 | 2 | 1 | IB2iii & IB2vi & *IB4ia (simple -cutaneous- syndactyly)* | 2 | A19. Greig cephalopolysyndactyly |
| IB1iv (Cleft hand) & IA2v (Radioulnar synostosis) | 2 | 2 | 1 | IB1iv & IA2v & *IA2vi (Congenital dislocation of radial head)* |  |  |
| IB4ia (simple -cutaneous- syndactyly) & IB4iiib (Synpolydactyly) | 2 | 1 | 1 | IB4ia & IB4iiib & *IB2vi (Ulnar polydactyly) & IB4iib (Clinodactyly)* |  | A35. Pallister-Hall |
| IB2i (Radial longitudinal deficiency, hypoplastic thumb) & IB4iib (Clinodactyly) | 2 | 2 | 1 | IB2i & IB4iib & *IB2ii (ulnar longitudinal defeciency, hypoplastic ulnar ray)* | 1 | A9. Catel-Manzke |
| IB4iiib (Synpolydactyly) & IB2vi (Ulnar polydactyly) | 2 | 1 | 1 | IB4iiib & IB2vi & *IB4ia (Simple -cutaneous- syndactyly) & IB4iib (Clinodactyly)* |  | A35. Pallister-Hall |
| IIICia (Arthrogryposis multiplex congenita - Amyoplasia) & IB2vi (Ulnar polydactyly) | 2 | 2 | 0 |  |  |  |
| IB2ii (ulnar longitudinal defeciency, hypoplastic ulnar ray) & IB2vi (Ulnar polydactyly) | 2 | 2 | 1 | IB2ii & IB2vi & *IA2ii (Ulnar longitudinal deficiency)* | 1 | A49. Ulnar-mammary |
| IB2iv (Thiphalangeal thumb) & IB2iva (Triphalangeal thumb -five finger hand-) | 2 | 1 | 1 | IB2iv & IB2iva & IB2iii |  |  |
| IB4iiic (Unspecified axis -complex, not otherwise specified-) & IA2ii (ulnar longitudinal deficiency entire upper limb) | 1 | 1 | 0 |  |  |  |
| IB1iv (Cleft hand) & IB4ia (Simple -cutaneous- syndactyly) | 1 | 1 | 0 |  |  |  |
| IB2iii (radial polydactyly) & IB4iiib (Synpolydactyly) | 1 | 1 | 0 |  | 1 | B. Others: Branchio-oculo-faciaal syndroom |
| IB2ii (ulnar longitudinal defeciency, hypoplastic ulnar ray) & IB4ia (Simple -cutaneous- syndactyly) | 1 | 1 | 0 |  |  |  |
| IB2v (ulnar dimelia -mirror hand) & IB2iii (Ulnar polydactyly) | 1 | 1 | 0 |  |  |  |
| IB2ii (ulnar longitudinal defeciency, hypoplastic ulnar ray) & IB4iiib (Synpolydactyly) | 1 | 1 | 0 |  |  |  |
| IB4ia (simple -cutaneous- syndactyly) & IA4ia (Sprengel deformity) | 1 | 1 | 0 |  |  |  |
| IB2i (Radial longitudinal deficiency, hypoplastic thumb) & IIICiia (Camptodactyly) | 1 | 1 | 0 |  |  |  |
| IB2i (Radial longitudinal deficiency, hypoplastic thumb) & IB2vi (Ulnar polydactyly) | 1 | 1 | 0 |  |  |  |
| IB2ii (ulnar longitudinal defeciency, hypoplastic ulnar ray) & IB4iiic (Unspecified axis -complex, not otherwise specified-) | 1 | 1 | 0 |  |  |  |
| IB1ii (Symbrachydactyly) & IA2v (Radioulnar synostosis) | 1 | 1 | 0 |  |  |  |
| IB4iiic (Unspecified axis -complex, not otherwise specified-) & IB2i (Radial longitudinal deficiency, hypoplastic thumb) | 1 | 1 | 0 |  |  |  |
| IB1ii (Symbrachydactyly) & IB1i (Brachydactyly) | 1 | 1 | 0 |  |  |  |
| IB1ii (Symbrachydactyly) &IIIB1i (Hemangioma) | 1 | 1 | 0 |  |  |  |
| IA2i (Radial longitudinal deficiency) & IB4ia (Simple -cutaneous- syndactyly) | 1 | 1 | 0 |  |  |  |
| IB4iid (Synostosis/symphalangism) & IB1i (Brachydactyly) | 1 | 1 | 0 |  |  |  |
| IA2vi (Congenital dislocation of radial head) & IIICic (Arthrogryposis multiplex congenita -other) | 1 | 1 | 0 |  | 1 | B. Others: Syndroom van Aarskog |
| IB2vi (Ulnar polydactyly) & IIICiib (Thumb in palm deformity) | 1 | 1 | 0 |  |  |  |
| IA2ii (Ulnar longitudinal deficiency entire upper limb) & IA2iv (Radiohumeral synostosis) | 1 | 1 | 0 |  |  |  |
| IB2i (Radial longitudinal deficiency, hypoplastic thumb) & IB2iv (Triphalangeal thumb) | 1 | 0 | 0 |  |  |  |
| IB2ii (ulnar longitudinal defeciency, hypoplastic ulnar ray) & IB1i (Brachydactyly) | 1 | 1 | 0 |  |  |  |
| IB2vi (Ulnar polydactyly) & IB4iib (Clinodactyly) | 1 | 1 | 0 |  |  |  |
| IB4iiib (Synpolydactyly) & IB4iib (Clinodactyly) | 1 | 1 | 0 |  |  |  |
| IB2iii (Radial polydactyly) & IB4ia (simple -cutaneous- syndactyly) | 1 | 1 | 1 | IB2iii & *IB2vi (Ulnar polydactyly)* & IB4ia |  |  |
| IB4ia (simple -cutaneous- syndactyly) & IB2i (Radial longitudinal deficiency, hypoplastic thumb) | 1 | 1 | 1 | IB4ia & *IB1i (Brachydactyly)* & IB2i |  |  |
| IB1i (Brachydactyly) & IB2i (Radial longitudinal deficiency, hypoplastic thumb) | 1 | 1 | 1 | *IB4ia (simple -cutaneous- syndactyly)* & IB1i & IB2i |  |  |
| IB1iv (Cleft hand) & IB4ia (simple -cutaneous- syndactyly) | 1 | 1 | 1 | IB1iv & IB4ia & *IB4iib (Clinodactyly)* |  |  |
| IB1iv (Cleft hand) & IB4iib (Clinodactyly) | 1 | 1 | 1 | IB1iv & *IB4ia (simple -cutaneous- syndactyly)* & IB4iib |  |  |
| IB1iv (Cleft hand) & IA2vi (Congenital dislocation of radial head) | 1 | 1 | 1 | IB1iv & IA2vi & *IA2v (Radioulnar synostosis)* |  |  |
| IA2v (Radioulnar synostosis) & IA2vi (Congenital dislocation of radial head) | 1 | 1 | 1 | *IB1iv (Cleft hand)* & IA2vi & IA2v |  |  |
| IB2i (Radial longitudinal deficiency, hypoplastic thumb) & IB2ii (ulnar longitudinal defeciency, hypoplastic ulnar ray) | 1 | 1 | 1 | IB2i & IB2ii & *IB4iib (Clinodactyly)* |  |  |
| IB4iib (Clinodactyly) & IB2ii (ulnar longitudinal defeciency, hypoplastic ulnar ray) | 1 | 1 | 1 | *IB2i (Radial longitudinal deficiency, hypoplastic thumb)* & IB2ii & IB4iib |  |  |
| IB2i (Radial longitudinal deficiency, hypoplastic thumb) & IB4ia (simple -cutaneous- syndactyly) | 1 | 1 | 1 | IB2i & IB4ia & *IA2vi (Congenital dislocation of radial head)* |  |  |
| IB2i (Radial longitudinal deficiency, hypoplastic thumb) & IA2vi (Congenital dislocation of radial head) | 1 | 1 | 1 | IB2i & *IB4ia (simple -cutaneous- syndactyly)* & IA2vi |  |  |
| IB4ia (simple -cutaneous- syndactyly) & IA2vi (Congenital dislocation of radial head) | 1 | 1 | 1 | *IB2i (Radial longitudinal deficiency, hypoplastic thumb)* & IB4ia & IA2vi | 1 | A22. Holt-Oram |
| IB2ii (ulnar longitudinal defeciency, hypoplastic ulnar ray) & IA2ii (Ulnar longitudinal deficiency) | 1 | 0 | 1 | IB2ii & IA2ii & *IB2vi (Ulnar polydactyly)* |  |  |
| IB2vi (Ulnar polydactyly) & IA2ii (Ulnar longitudinal deficiency) | 1 | 0 | 1 | *IB2ii (ulnar longitudinal defeciency, hypoplastic ulnar ray)* & IA2ii & IB2vi |  |  |
| IA2i (Radial longitudinal deficiency) & IA2v (Radioulnar synostosis) | 1 | 1 | 1 | IA2i & IA2v & *IB4iib (Clinodactyly)* |  |  |
| IA2i (Radial longitudinal deficiency) & IB4iib (Clinodactyly) | 1 | 1 | 1 | IA2i & *IA2v (Radioulnar synostosis)* & IB4iib |  |  |
| IA2v (Radioulnar synostosis) & IB4iib (Clinodactyly) | 1 | 1 | 1 | *IA2i (Radial longitudinal deficiency)* & IA2v & IB4iib |  |  |
| IB1iv (Cleft hand) & IB2iv (Triphalangeal thumb) | 1 | 1 | 1 | IB1iv & IB2iv & *IB4iia (Osseous -complex- syndactyly)* |  |  |
| IB1iv (Cleft hand) & IB4iia (Osseous -complex- syndactyly) | 1 | 1 | 1 | IB1iv & *IB2iv (Triphalangeal thumb)* & IB4iia | 1 | A37. Pierre Robin |
| IB2iv (Thiphalangeal thumb) & IB2iii (Radial polydactyly) | 1 | 1 | 1 | IB2iv & IB2iva & IB2iii |  |  |
| IB2iva (Thiphalangeal thumb) & IB2iii (Radial polydactyly) | 1 | 1 | 1 | IB2iv & IB2iva & IB2iii |  |  |
| IB2iv (Triphalangeal thumb) & IB4iia (Osseous -complex- syndactyly) | 1 | 0 | 1 | *IB1iv (Cleft hand)* & IB2iv & IB4iia |  |  |
| Total (61 different combinations) | 89 | 77 | 32 |  | 11 |  |
